# Novel copy number variations within *SYCE1* caused meiotic arrest and non-obstructive azoospermia

**DOI:** 10.1186/s12920-022-01288-8

**Published:** 2022-06-19

**Authors:** Yuhua Huang, Ruhui Tian, Junwei Xu, Zhiyong Ji, Yuxiang Zhang, Liangyu Zhao, Chao Yang, Peng Li, Erlei Zhi, Haowei Bai, Sha Han, Jiaqiang Luo, Jingpeng Zhao, Jing Zhang, Zhi Zhou, Zheng Li, Chencheng Yao

**Affiliations:** 1 Department of Andrology, Shanghai Key Laboratory of Reproductive Medicine, The Center for Men’s Health, Urologic Medical Center, Shanghai General Hospital, Shanghai Jiao Tong University, Shanghai, 200080 China; 2 School of Life Science and Technology, ShanghaiTech University, Shanghai, 201210 China; 3 State Key Lab of Reproductive Medicine, Nanjing Medical University, Nanjing, 211166 China; 4 Reproductive Medicine Research Center, The Sixth Affiliated Hospital of Sun Yat-Sen University, Guangzhou, 510620 China

**Keywords:** CNVs, Meiosis, Azoospermia, Gene mutations, Spermatogenesis

## Abstract

**Background:**

Non-obstructive azoospermia (NOA) is the most severe disease in male infertility, but the genetic causes for majority of NOA remain unknown.

**Methods:**

Two Chinese NOA-affected patients were recruited to identify the genetic causal factor of infertility. Whole-exome sequencing (WES) was conducted in the two patients with NOA. Sanger sequencing and CNV array were used to ascertain the WES results. Hematoxylin and eosin (H&E) staining and immunofluorescence (IF) were carried out to evaluate the stage of spermatogenesis arrested in the affected cases.

**Results:**

Novel heterozygous deletion (LOH) within *SYCE1* (seq[GRCh37] del(10)(10q26.3)chr10:g.135111754_135427143del) and heterozygous loss of function (LoF) variant in *SYCE1* (NM_001143763: c.689_690 del:p.F230fs) were identified in one NOA-affected patient. While homozygous deletion within *SYCE1* (seq[GRCh37] del(10)(10q26.3)chr10:g.135340247_135379115del) was detected in the other patient with meiotic arrest. H&E and IF staining demonstrated that the spermatogenesis was arrested at pachytene stage in the two patients with NOA, suggesting these two novel CNVs within *SYCE1* could lead to meiotic defect and NOA.

**Conclusions:**

We identified that two novel CNVs within *SYCE1* are associated with meiotic arrest and male infertility. Thus, our study expands the knowledge of variants in *SYCE1* and provides a new insight to understand the genetic etiologies of NOA.

**Supplementary Information:**

The online version contains supplementary material available at 10.1186/s12920-022-01288-8.

## Background

Infertility affects about 15% of couples worldwide and one in eight couples encounter problems when attempting to conceive the first child. And a male-infertility-associated factor could be found in approximately half of all couples. Azoospermia, which is defined as the complete absence of spermatozoa in the ejaculate, accounts for 10% ~ 15% of male infertility cases. 70% azoospermic cases represent non-obstructive azoospermia (NOA) with the absence or reduction of germ cells owing to the testicular atrophy. Based on testis biopsy and subsequent pathologic analysis, NOA could be classified into three types, including Sertoli cell only syndrome (SCOS), maturation arrest (MA) and hypo-spermatogenesis (HS). MA is characterized by the presence of germ cells that do not complete spermatogenic development, most of which is meiotic arrest. It was showed that complete germ cell arrest occurs at the spermatocyte period in 12% azoospermic men [1].

Genetic etiology has been revealed for meiotic defects in recent decades. It is illustrated that translocation between autosome and sex chromosome was associated with meiotic defects mainly due to the chromosomal pairing disturbances around the breakpoints [2–4]. Micro-deletions of Y chromosome could result in meiotic defects mainly because of deficiency in RNA Binding Motif Protein X-Linked (*RBMY*), the RNA binding protein located in Y chromosome [5–7]. Furthermore, through whole-exome sequencing (WES) of NOA pedigree study, several single-nucleotide variants (SNVs) and inDels have been identified as the cause of meiotic defects in human, including DNA Meiotic Recombinase 1 (*DMC1* MIM: 602721), Stromal Antigen 3 (*STAG3* MIM:608489), Testis Expressed 11 (*TEX11* MIM: 300311), Shortage In Chiasmata 1 (*SHOC1*, MIM: 618038), Synaptonemal Complex Central Element Protein 1 (*SYCE1* MIM: 611486), Meiosis Specific With OB-Fold (*MEIOB* MIM: 617670), Coiled-Coil Domain Containing 155 (*CCDC155* MIM: 618125), Testis Expressed 14 (*TEX14* MIM: 605792), Testis Expressed 15 (*TEX15* MIM: 605795), and X-Ray Repair Cross Complementing 2 (*XRCC2* MIM: 600375) [8–16]. Recent years have seen the emergence of copy number variation (CNV) as an important source of genetic diversity. CNVs are defined as submicroscopic chromosomal deletions, insertions or duplications in the human genome ranging from 50 bp to several Mb [17]. It is estimated that approximately 5–10% of the human genome contributed to CNVs. Most CNVs are benign, however, maladaptive CNVs are associated with common or rare genetic disorders, such as autism [18], type 1 diabetes [19], Charcot-Marie-Tooth disease type 1A [20], and hemophilia A [21]. CNVs were also associated with meiotic arrest. It is illustrated that eight deletions/duplications might be linked with maturation arrest [22]. Moreover, it is identified that heterozygous duplication in *MAST2*, *MYRIP*, *LRRC4C* and the long noncoding RNA *LOC100507205* are associated with meiotic arrest [23, 24]. However, the roles of MAST2, MYRIP, LRRC4C and the long noncoding RNA *LOC100507205* were unknown in the germ cell development. It was illustrated that a homozygous deletion of the entire open reading frame of the *SYCE1* gene has been reported in a sporadic NOA patient [25]. Also, Huang et al. identified one homozygous CNV within 134-kb deletion on chromosome 10 encompassing the *SYCE1* gene in one azoospermic man, suggesting *SYCE1* is located at rearrangement hotspot [23]. However, many other types of CNVs in *SYCE1* which were associated with NOA remain to be elucidated. Also, the association between CNVs with *SYCE1* and histopathology still remains unclear.

Herein, we identified two novel CNVs within *SYCE1* in two NOA-affected patients. We identified heterozygous deletion within *SYCE1* associated with heterozygous LoF variant in *SYCE1* in one patient with NOA and a homozygous deletion within *SYCE1* in the other patient with meiotic arrest. Hematoxylin and eosin staining (H&E) and immunofluorescence (IF) showed that the spermatogenesis arrested at spermatocyte stage in the two NOA-cases. Thus, our study ascertained CNVs within *SYCE1* could result in meiotic arrest and NOA, and provided novel foci for NOA genetic counselling.

## Methods

### Study subjects

In the current study, two Chinese NOA-affected patients were recruited to identify the genetic causal factor of male infertility at our center. The family histories of two patients with NOA were collected. The known causal factors for male infertility were excluded for the two NOA-affected individuals, namely cryptorchidism, hypogonadism, chromosomal abnormalities, genomic AZF micro-deletions, seminal tract obstruction, and other diseases like cancer.

### Whole-exome sequencing (WES)

Genomic DNA was extracted from blood samples of two NOA-affected patients using the TIANamp Blood DNA Kit (Tiangen). WES of samples was prepared by VAHTS Universal DNA Library Prep Kit for Illumina V3 (Vazyme) and IDT xGen Exome Research Panel V1.0 (Integrated DNA Technologies). The quantity of sequencing library was assessed by Qubit 3.0 fluorometer (Thermo Fisher Scientific). The quality and size of libraries were measured by 2100 Bioanalyzer High Sensitivity DNA Assay (Agilent Technologies). For next-generation sequencing, the qualified libraries were applied to 2 × 150-bp paired-end sequencing on the Illumina NovaSeq platform (Illumina, San Diego, USA).

### In silico analysis

Raw data files were obtained from Novaseq 6000, and then were demultiplexed and converted to fastq format using bcl2fastq software for downstream analysis. Adapters and reads with low quality were trimmed using fastp software. The BAM files were obtained by aligning the sequence reads to the reference (hg19/GRCH37, fasta format) with the use of the SpeedSeq. Additionally, duplicate reads were flagged in the BAM files to prevent downstream variant call errors, sample contamination and swaps using VerifyBamID. Then UnifiedGenotyper tool of GAT was used to call SNVs. The variants were annotated using Annovar software. During the annotation, several public databases such as Clinvar, gnomAD, dbNSFP, etc. were used. Variants with allele frequencies higher than 1% in any public databases (ExAC Browser and gnomAD) were excluded. Because autosomal recessive or X-linked inheritance were assumed for MA, genes with two alleles of potentially deleterious missense mutations (SIFT, PolyPhen-2 and MutationTaster), LoF mutations, or CNVs were kept for further analysis. Moreover, we compared candidate genes with human testis-enriched genes in the database (https://www.proteinatlas.org/) and known pathogenic genes for azoospermia in mice (http://www.informatics.jax.org/mgihome/homepages/).

### Sanger sequencing

Validation of *SYCE1* SNV in the NOA-affected patient (P6326) and family members was performed by classical Sanger sequencing. Genomic DNA was extracted from peripheral blood using the TIANamp Genomic DNA Blood Kit (TIANGEN, Beijing, China) according to the manufacturer’s instructions. The primers were shown as follows: Forward primer: 5′-CAGAGATGTGGGATGACAGAAG-3′; Reverse primer: 5′-AGAAGGTGGAGAGAGGAGATAC-3′. And the PCR products were bidirectionally sequenced through a 3730xl DNA Analyzer (Applied Biosystems, California, USA).

### CNV array

CNV array was performed according to the method as described previously [26]. Briefly, for each blood sample of two patients with NOA and the family members, 250 ng genomic DNA was digested by NspI nuclease for 2 h at 37 °C. Digested DNA and adaptors were ligated by T4 DNA ligase for 3 h at 16 °C. Ligated DNA was amplified, fragmented, end-labeled with biotin and then hybridized to an Affymetrix CytoScan HD Array. Arrays were incubated at 50 °C for 16 h in a Hybridization Oven 645 with rotary motion (60 rpm), followed by washed and stained in a Fluidics Station 450 with protocol “CytoScanHD_Array_450” and scanned with scanner 3000 7 G controlled by Affymetrix GeneChip Command Console Software (AGCC v4.0.0). Raw data were analyzed by Chromosome Analysis Suite (ChAS) Software v2.1 and copy number was determined by the Affymetrix CytoScanHD REF model. All microarray experiments were carried out by the CytoScan HD Array Kit and Reagent Kit Bundle (catalogue number: 901835) following the manufacturer’s protocol.

### Hematoxylin and eosin staining (H&E staining)

The testicular tissues of the two NOA-affected individuals were fixed in 4% paraformaldehyde solution overnight, embedded in paraffin and sectioned at 5 μm thickness. The sections were then stained with Hematoxylin and Eosin solution (catalogue number: ab245880, Abcam, Cambridge, UK) according to standard protocols. The images were captured by phase-contrast microscope (Leica).

### Immunofluorescence (IF)

Immunofluorescence was performed according to the method as described previously [8]. In brief, the testicular biopsies were obtained from the two patients with NOA. The testicular tissue was fixed overnight in 4% paraformaldehyde at 4 °C, and then embedded in warm paraffin (60 °C). The biopsies were sectioned at 5 μm thickness. The tissue sections were dewaxed in xylene, re-hydrated in a descending alcohol gradient, and heated in sodium citrate buffer (90–98 °C) for 15 min for antigen retrieval. After blocking with 5% BSA for 1 h at room temperature, the sections were incubated overnight with anti-SYCP3 (dilution: 1:25; catalogue number: AF3750, R&D Systems), anti-γH2AX (dilution: 1:300; catalogue number: 2668445, Millipore), anti-DMC1 (dilution: 1:100; catalogue number: sc-373862, Santa Cruz) and PNA (dilution: 1:400; catalogue number: L21409, Thermo Fisher Scientific) at 4 °C. The sections were washed thrice with PBS-T (Phosphate buffer saline-Tween), and incubated with highly cross-adsorbed secondary antibody conjugated with Alexa Fluor® 488 or Alexa Fluor® 594 (dilution: 1:400; Thermo Fisher Scientific) for 1 h at room temperature. The sections were washed three times with PBST and counterstained with 4’,6-diamidino-2-phenylindole (DAPI) to label the nuclei. The images were captured by fluorescence microscope (Leica).

## Results

### Clinical data

**Fig. 1:**
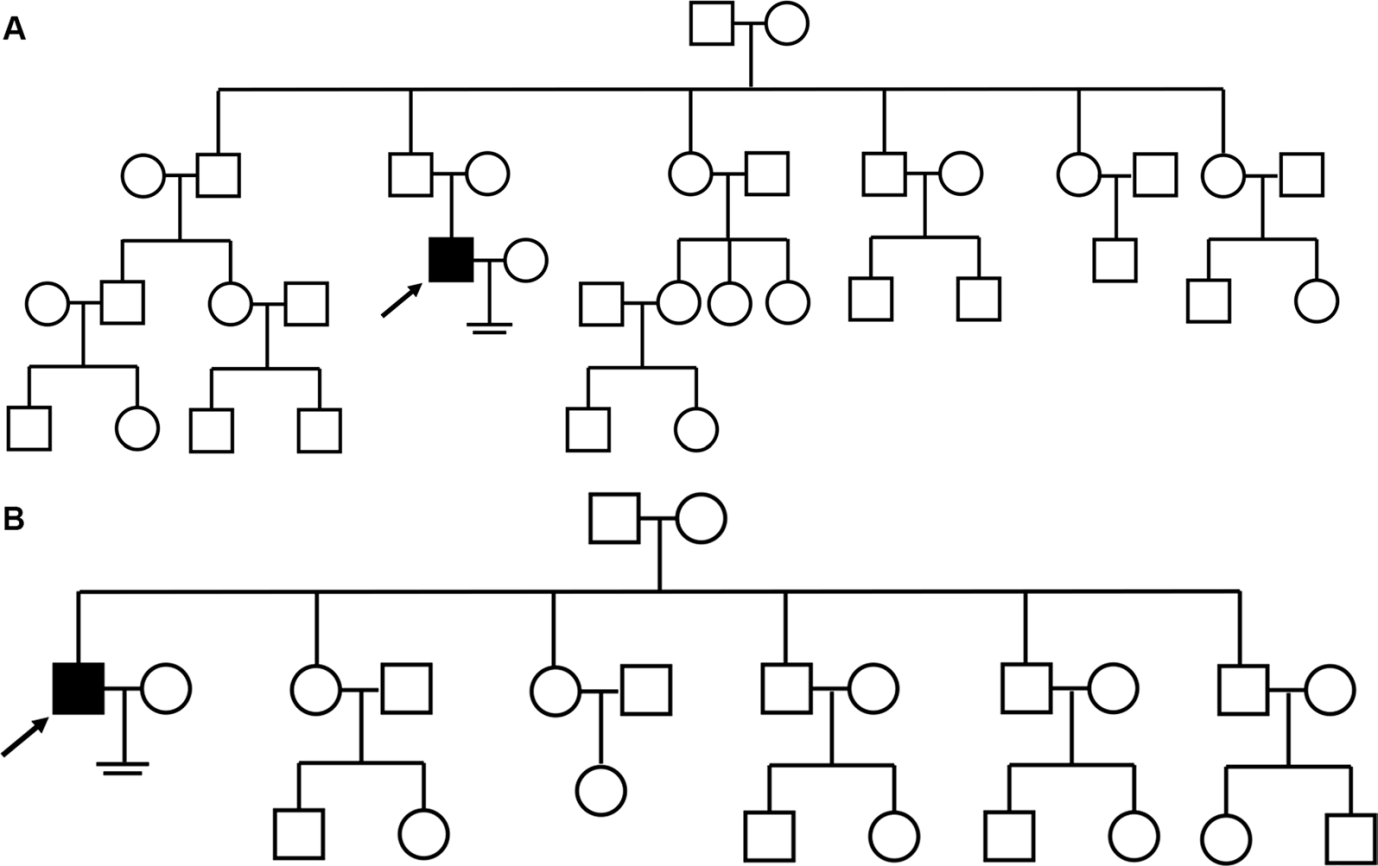
Pedigree of the NOA-affected patients. **A**–**B** Family pedigree with black fill denoting NOA-affected patient P6326 (**A**) and P10377 (**B**). The arrowheads indicate the probands (P6326 and P10377)

Two Chinese patients with infertility participated in this study. The proband (P6326) had a history of male infertility for 5 years. And primary infertility was observed in the other proband (P10377). There was no family history of consanguinity or fertility problems and no chronic diseases in the two NOA-affected patients (Fig. 1A and B). Neither patient had a history of cryptorchidism, hypogonadism, cancer, drinking, or smoking. Physical examination revealed normal development of penis, epididymis, prostate, scrotum, and vas deferens. Also, there was no varicocele in the two patients with NOA. The volumes of testes in patients (P6326 and P10377) were 15 ml (both sides) and 12 ml (both sides) respectively. Semen analysis revealed normal volume and complete azoospermia on basis of the WHO guidelines (5th edition). Laboratory examination showed that sex hormone levels in both patients were comparable to the reference values (Table 1). Both had 46, XY karyotypes and there were no microdeletions in the Y chromosome.

**Table 1:** Clinical characteristics of the NOA-affected patients

	P6326	P10377	Reference
Age at phenotyping	30	30	/
Height (cm)	178	180	/
Weight (kg)	90	72	/
Karyotype	46, XY	46, XY	46, XY
Y chromosome microdeletions	Normal	Normal	Normal
Semen analysis			
Semen volume (mL)	3	4	≥ 1.5
Concentration (millions/mL)	0	0	≥ 15
PR (%)	0	0	≥ 32
NP (%)	0	0	/
IM (%)	0	0	/
Centrifuged spermatozoa number (/ejaculate)	0	0	/
Hormone analysis			
FSH (IU/L)	4.53	4.26	1.27–19.26
LH (IU/L)	6.67	3.38	1.24–8.62
E2 (pg/mL)	8.22	27	< 38.95
T (μg/L)	6.33	2.63	1.75–7.81
P (ng/mL)	7.73	9.61	2.64–13.13

*PR* progressive, *NP* non-progressive, *IM* immotility, *FSH* follicle-stimulating hormone, *LH* luteinizing hormone, *E2* estradiol, *T* testosterone, *P* Prolactin

The probands in families 1 and 2 underwent microsurgical testicular sperm extraction (mTESE) at our center. Histopathological analysis revealed that the Johnsen score of both NOA-affected patients was 5, suggesting MA phenotype in both patients.

### Identification of *SYCE1* pathogenic variants in the NOA-affected patients

**Fig. 2:**
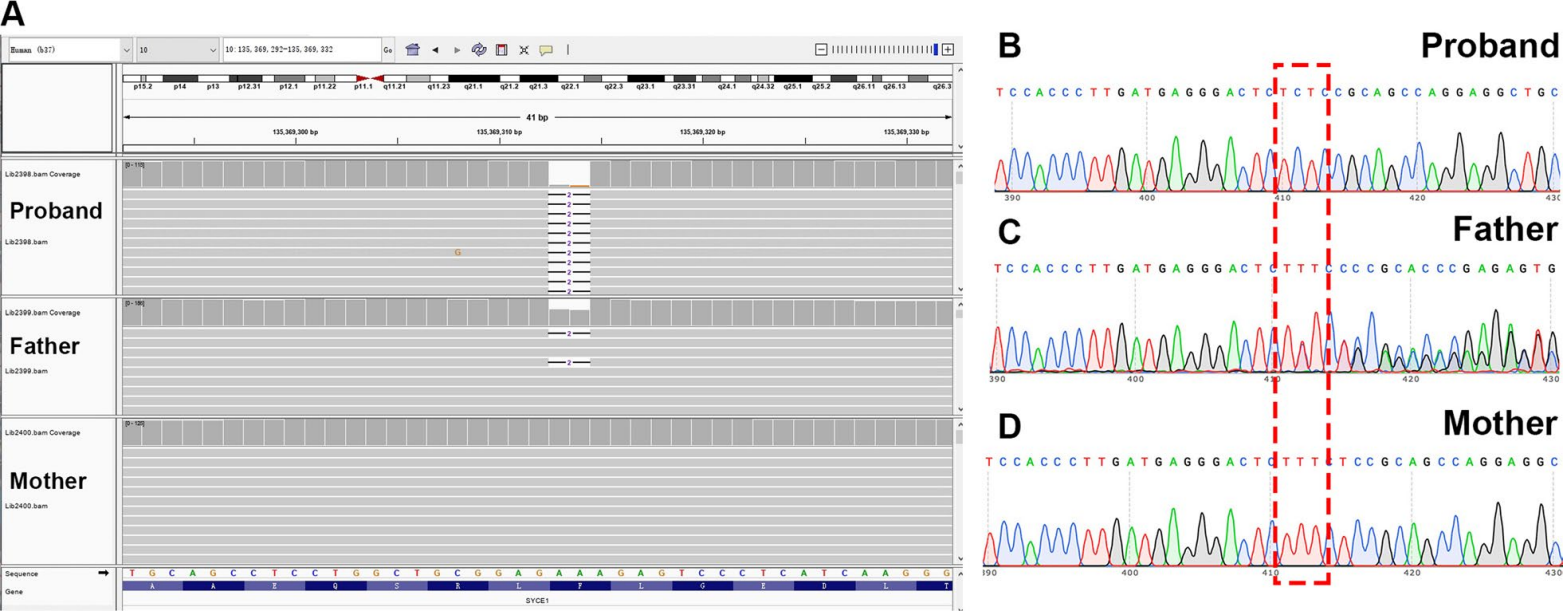
WES and Sanger sequencing of *SYCE1* SNV in the NOA-affected patient. **A** Blood samples from the NOA-affected patient and his parents were detected with WES. Frameshift variant (F230fs) was identified in the proband and his father. The maternal allele appears to be normal. **B**–**D** Validation of *SYCE1* SNV identified in WES using Sanger sequencing in the patient (P6326) (**B**) and his parents (**C** and **D**)

**Fig. 3:**
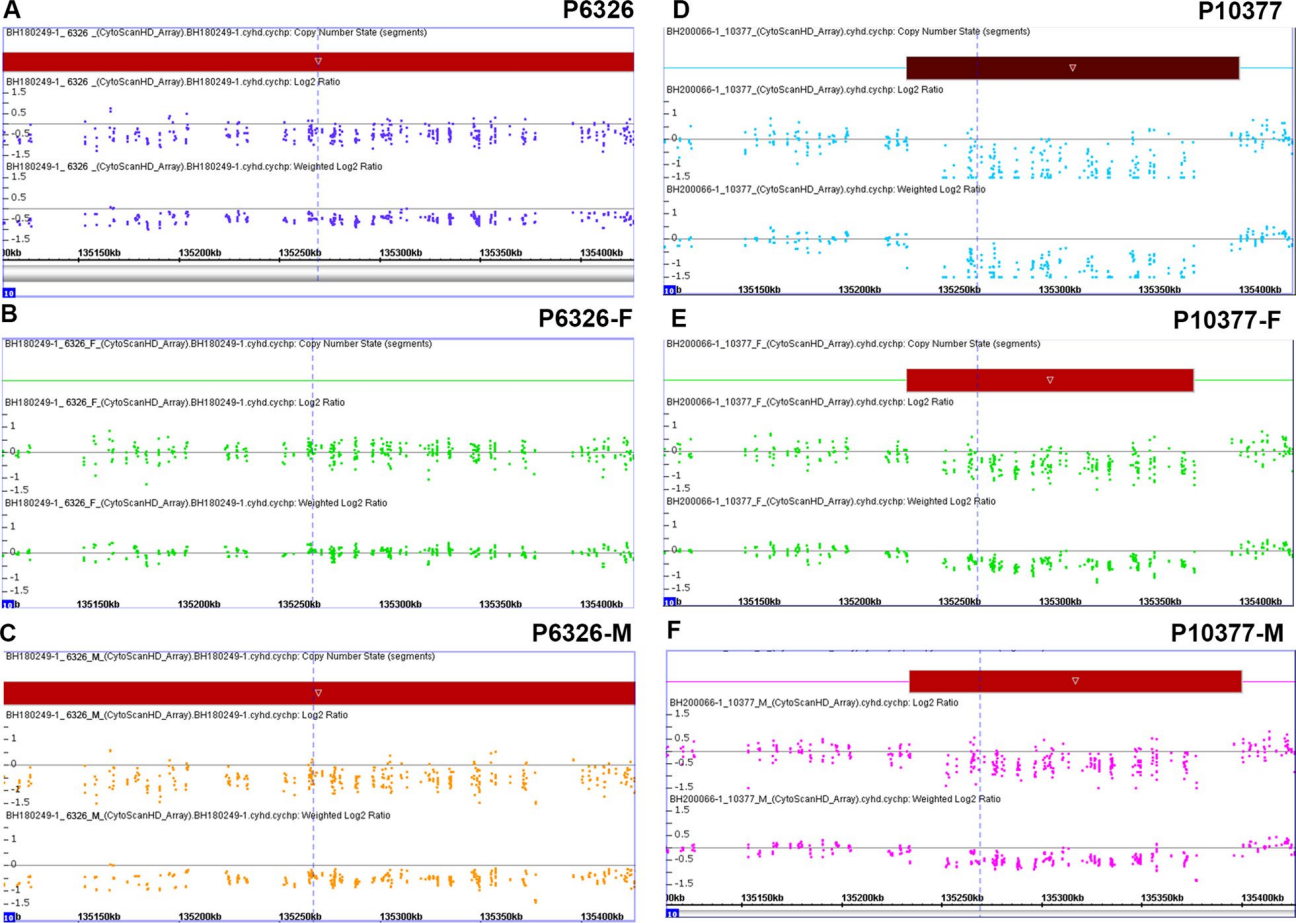
CNVs within *SYCE1* identified in the NOA-affected family using CNV array. **A**–**C** CNV array revealed the CNV (seq[GRCh37] del(10)(10q26.3)chr10:g.135111754_135427143del) in the proband (P6326) (**A**), father (**B**) and mother (**C**); **D**–**F** CNV array revealed the CNV (seq[GRCh37] del(10)(10q26.3)chr10:g.135340247_135379115del) in the NOA-affected patient (P10377) (**D**), father (**E**) and mother (**F**)

WES assay were performed on the two NOA-affected patients or their family members. After the genetic analyses pipeline aforementioned in the methods, LoF variant (NM_001143763: c.689_690 del:p.F230fs) in *SYCE1* was identified using WES and Sanger sequencing (Fig. 2A and B) in the NOA-affected patient (P6326). The heterozygous frameshift variant (F230fs) was identified in the father (Fig. 2A and C). However, the maternal allele appears to be normal (Fig. 2A and D). It was possible that the proband carried a heterozygous F230fs mutation on the paternal allele and a heterozygous deletion in SYCE1 on the maternal allele. Thus, we employed the WES for CNV analysis in this case according to the protocol as described previously [27, 28]. Intriguingly, there seemed to be CNV in *SYCE1* in the proband and the mother (Additional file 1: Fig. 1A–C). Furthermore, CNV array verified that heterozygous deletion (LOH) within *SYCE1* (seq[GRCh37] del(10)(10q26.3)chr10:g.135111754_135427143del) was identified in the NOA-affected patient (P6326) and his mother but not in his father (Fig. 3A–C). Moreover, to evaluate whether the LoF variant (F230fs) are responsible of a reduced protein expression, we generated the mutant SYCE1 (SYCE1-MUT) coding DNA sequence (CDS) cloned downstream of the Flag tag (Flag-SYCE1-MUT). Western blot assay showed a truncated fusion protein, and the expression of mutant SYCE1 was significantly decreased compared with wide type (Flag-SYCE1-WT) (Additional file 1: Fig. 1D). Thus, a paternally inherited frameshift variant led to a truncated SYCE1 protein with reduced expression. And maternally inherited CNV (seq[GRCh37] del(10)(10q26.3)chr10:g.135111754_135427143del) resulted in defect of SYCE1 protein expression in the NOA-affected proband (P6326).

For the patient with NOA (P10377), homozygous deletion within *SYCE1* (seq[GRCh37] del(10)(10q26.3)chr10:g.135340247_135379115del) was detected via CNV array. Consistent with the autosomal recessive mode of inheritance, the unaffected parents were heterozygous carriers of this same CNV variant (Fig. 3D–F). Collectively, heterozygous LoF SNV associated with heterozygous CNV within *SYCE1* were identified in the patient (P6326) and homozygous CNV within *SYCE1* was detected in the patient (P10377).

### MA Phenotypes in the patients with *SYCE1* CNVs

**Fig. 4:**
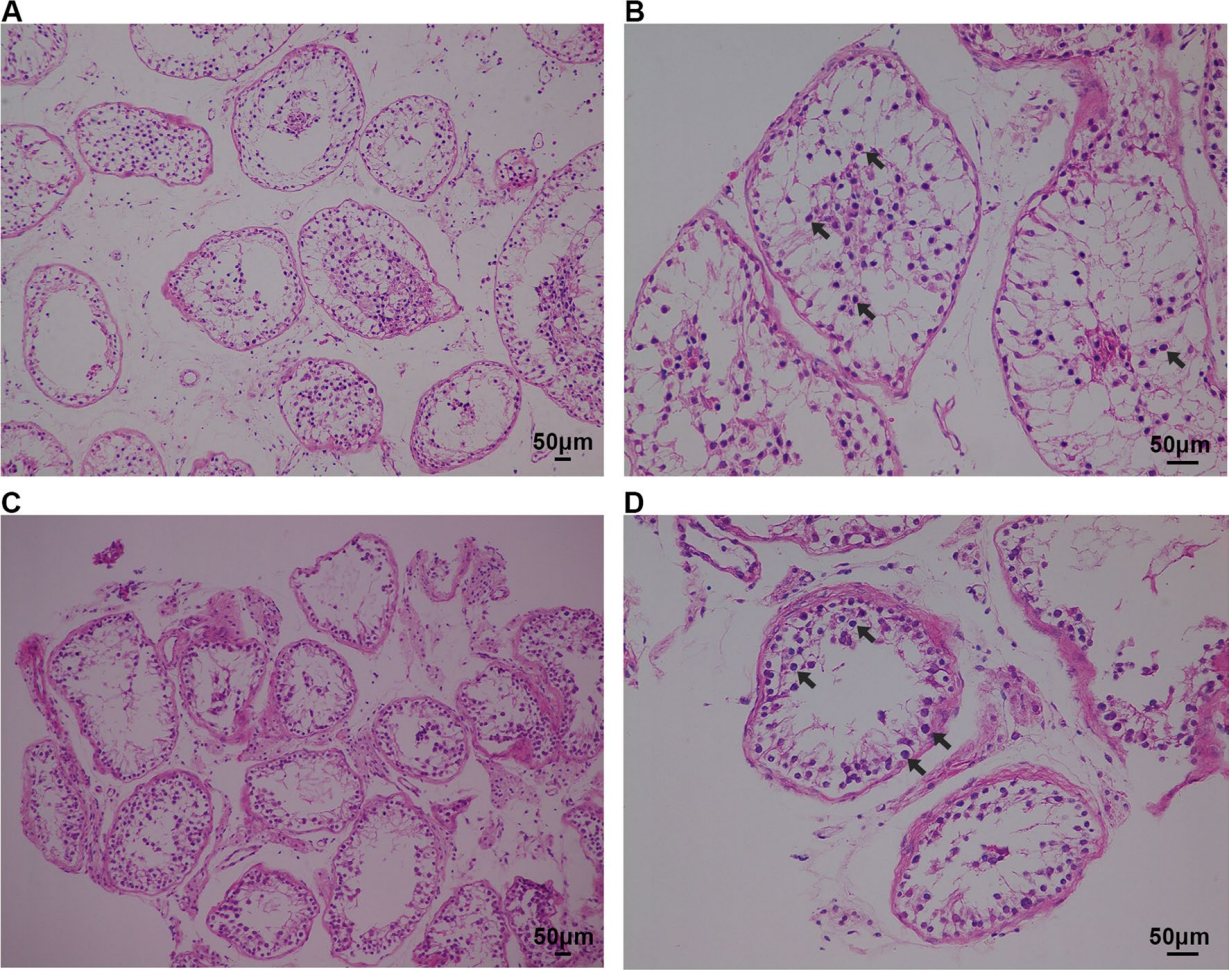
H&E staining of cross-sections of testis in NOA-affected patients. (**A**–**B**) H&E staining of cross-sections of testicular biopsy in the patient with NOA (P6326). (**C**–**D**) H&E staining of cross-sections of seminiferous tubules in the NOA-affected patient (P10377). Scale bars = 50 μm in **A–D**. And arrows indicated the spermatocytes in the testis

**Fig. 5:**
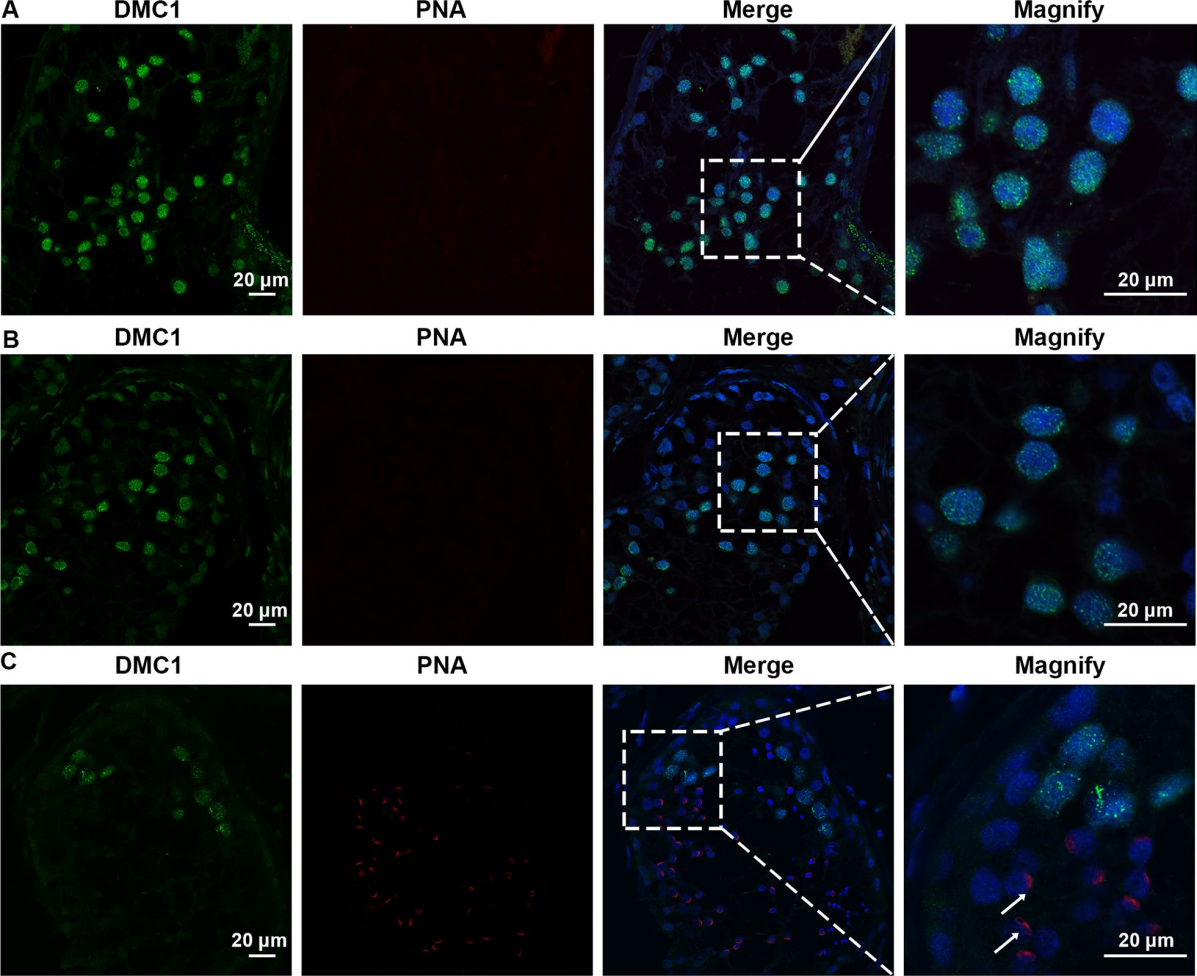
Expression of DMC1 and PNA in the testes of NOA-affected patients and patient with OA as a positive control. **A**–**C** Immunofluorescence staining showed the expression of DMC1 (green) and PNA (red) in the testicular tissue of NOA-affected patient P6326 (**A**), P10377 (**B**), and the patient with OA as a positive control (**C**). Scale bars = 20 μm in **A**–**C**. And the arrow indicates the acrosome of spermatids in the testis

**Fig. 6:**
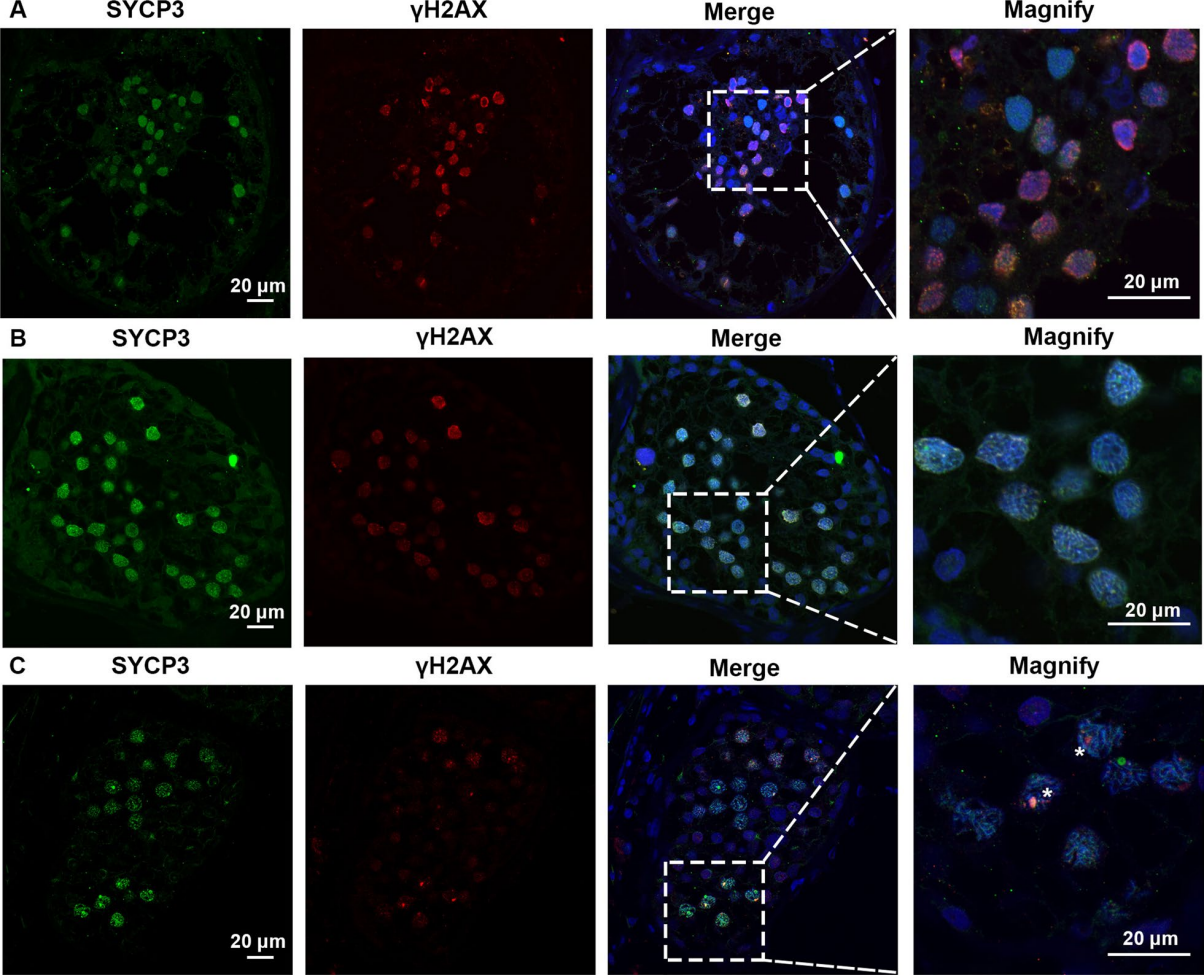
Expression of SYCP3 and γH2AX in the testis of the patient with *SYCE1* CNVs and OA-affected patient as a positive control. **A**–**C** Immunofluorescence staining showed the expression of SYCP3 (Green) and γH2AX (Red) in the testis of the patient P6326 (**A**), P10377 (**B**), and the patient with OA as a positive control (**C**) Scale bars = 20 μm in **A**–**D**. And the arrowhead indicated the XY body in the spermatocytes in the testis of the patient with OA

MA phenotypes in the NOA-affected male patients with CNVs within *SYCE1* were ascertained by H&E and IF staining. H&E staining results revealed that decreased number of spermatocytes and absence of spermatozoa and spermatids in the testes of the patients with *SYCE1* CNVs (P6326 and P10377). However, the number of spermatogonial stem cells (SSCs), differentiated spermatogonia and Sertoli cells at the basement membrane within the seminiferous tubules remained not significantly changed (Fig. 4A–D). IF revealed the expression of DMC1, a marker of double-strand break repair, in the seminiferous tubules, suggesting normal DNA double-stranded break (DSB) repair in the meiosis prophase I in these two patients (P6326 and P10377). However, no signal of PNA (a marker of spermatids and spermatozoa) was detected (Fig. 5A–B). The testicular tissue from OA patients with normal spermatogenesis was also evaluated. And PNA was specifically expressed in acrosome of the haploid germ cells (Fig. 5C). Moreover, SYCP3 and γH2AX foci were expressed in the testis of the NOA-affected patients (Fig. 6A–B). SYCP3 is a marker of components of the axial/lateral element (AE and LE), and γH2AX foci is used to label the DSB, both of which are expressed in preleptotene to zygotene spermatocytes of prophase I. γH2AX can be also used to label the XY body, a specialized meiotic chromatin domain in the nucleus of pachytene spermatocytes. Nevertheless, there was no expression of XY body in seminiferous tubules of these patients with NOA (Fig. 6A–B). And the spermatogenesis was arrested at pachytene stage according to the SYCP3 staining. In contrast, positive expression of SYCP3 and XY body which was indicated by γH2AX staining were observed in testis from the patient with OA (Fig. 6C). Altogether, these results indicated the spermatogenesis arrested at the pachytene stage in the two NOA-affected patients with *SYCE1* CNVs.

## Discussion

In the current study, we report novel CNVs within *SYCE1* in two azoospermic patients with meiotic defect. Heterozygous deletion (LOH) (seq[GRCh37] del(10)(10q26.3)chr10:g.135111754_135427143del) and heterozygous LoF variant (F230 fs) in *SYCE1* were identified in the NOA-affected patient (P6326). Also, homozygous deletion within *SYCE1* (seq[GRCh37] del(10)(10q26.3)chr10:g.135340247_135379115del) was detected in the NOA-affected patients (P10377). H&E and IF staining demonstrated that the spermatogenesis of both patients with NOA arrested at pachytene stage in prophase I. Thus, our study revealed that CNVs within *SYCE1* were associated with meiotic arrest and NOA.

CNV is the main type of structure variation (SV) caused by genomic rearrangement, which mainly includes deletion and duplication of sub-microscopic genomic segments ranging from 50 bp to several Mb. CNV has been recognized as one of the main genetic factors underlying human diseases. Rearrangement hotspots are the highly homologous regions within segmental duplications (SDs) which could influence rearrangement events. And they are considered antecedents to the formation of CNVs [29]. It was illustrated that 47 hotspots within *SYCE1* were detected in 970 Han Chinese men with NOA. However, only one deletion homozygote was identified [23]. Herein, we identified that two novel CNVs within *SYCE1* caused meiotic arrest and male infertility. There are no previous reports of CNV (seq[GRCh37] del(10)(10q26.3)chr10:g.135111754_135427143del), while allele frequencies of CNV (seq[GRCh37] del(10)(10q26.3)chr10:g.135340247_135379115del) was 6.1 × 10^–3^ according to the DGV database. The CNVs in these two patients with NOA were assessed as deleterious, including PVS1 (For Spermatogenic failure 15, LoF variant of *SYCE1* is a known mechanism, this variant is a gene deletion); PM2 (Absent from controls in Exome Sequencing Project,1000 Genomes Project, or Exome Aggregation Consortium); PM3 (For recessive disorders, detected in trans with a pathogenic variant) according to the American College of Medical Genetics and Genomics and the Association for Molecular Pathology (ACMG/AMP) guidelines (Table 2).

**Table 2:** SNV and CNVs of *SYCE1* in the subjects with meiotic defects

Position	ID	Gene	cDNA mutation	Protein alteration	gnomAD Dataset		DGV Dataset		Genotype		
					MAF	Carriers	MAF	Carriers	Case(s)	Father	Mother
SNV of *SYCE1* identified in NOA-affected patient (P6326) via WES											
clirlO: 135369312	rs777697888	SYCE1	c.689_690 del	p.F230 fs	1.2 × l0^5^	Het:3:Hom:0	**/**	**/**	G/G	GAA/G	GAA/GAA
CNVs of *SYCE1* identified in NOA-affected patients using CNV array											
chrlO: 135111754_ 135427143	NA	SYCE1	/	**/**	**/**	**/**	NA	NA	het	wt	het
clirlO: 135340247_ 135379115	gssvL16107	SYCE1	/	**/**	**/**	**/**	6.1 × l0^−3^	Het:97;Hom:0	hom	het	het

Previously, two homozygous splice site mutation in the *SYCE1* (c.197-2 A > G and c.375-2A > G) were identified in two patients with NOA [12, 30]. Furthermore, de Vries et al. reported a nonsense homozygous mutation in the *SYCE1* (c.613C > T) in primary ovarian insufficiency (POI) sisters [31]. Thus, deleterious mutation in *SYCE1* could result in NOA and POI. Recently, another homozygous mutation (R125G) in *SYCE1* was identified in one patient with maturation arrest [32]. Also, Feng et al.reported the same homozygous mutation (F230fs) in *SYCE1* in one NOA-affected patient, and the variant was inherited from heterozygous parental carriers [33]. In the present study, for the patient with NOA (P6326), frameshift variant (F230fs) was inherited from the father whilst the CNV is inherited from mother, which was also consistent with the autosomal recessive mode of inheritance. The allele frequency of *SYCE1* variant (F230fs) was 1.2 × 10^–5^ according to the gnomAD database. And this *SYCE1* variant was assessed as deleterious, including PVS1 (For Spermatogenic failure 15, LoF variant of *SYCE1* is a known mechanism, this variant is a frameshift variant); PM2 (For recessive disorders, this variant is at extremely low frequency in Exome Sequencing Project,1000 Genomes Project, or Exome Aggregation Consortium); PM3 (For recessive disorders, detected in trans with a pathogenic variant) according to the American College of Medical Genetics and Genomics and the Association for Molecular Pathology (ACMG/AMP) guidelines (Table 2).

The synaptonemal complex (SC) is a highly ordered meiosis-specific scaffold that assembles between homologous chromosomes in the meiosis (Prophase I) and is essential for the formation of meiotic crossovers [34]. And SC structure is conserved between different organisms, including budding yeast, plants, flies, mice, and human. SC at pachytene consists of two lateral elements and the central region, while the central region is comprised of the transverse filaments and the central element (CE). CE proteins have been categorized as synaptic initiation factors (SYCE3, SYCE1, and SIX6OS1) and elongation factors (SYCE2 and TEX12). SYCE1 is the major component of the central element, and it is a meiosis-specific gene located at 10q26 in humans, which has 13 exons encoding a 351-aa protein [35]. Knock-out of *Syce1* in mice results in complete loss of tripartite SC structure [36]. It was demonstrated that SYCE1 forms head-of-head antiparallel dimer via SYCE1 core (aa 25 to 179), and this dimer undergoes conformational change into 1:1 complex upon interaction with SIX6OS1 [35]. The interaction is formed via two interfaces, including SYCE1 core-SIX6OS1N (Interface 1) and SYCE1 177–305 and downstream sequence within SIX6OS1 1–262 (Interface 2) [37]. Furthermore, SYCE1 could interact with SYCE3 through C-terminal, which is essential for SC recruitment [38]. In the present study, paternal derived CNV in patient (P6326) leaded to haploinsufficiency in SYCE1. While maternal derived *SYCE1* variant (NM_001143763: c.689_690 del:p.F230 fs) resulted in a truncated protein without any effects of SYCE1 core expression. However, this variant blocked the second binding interface with SIX6OS1. And SYCE1-SYCE3 complex could be significantly abolished in this variant, which resulted in severer defects in CE loading. In the NOA-affected patient (P10377), homozygous deletion leaded to no expression of SYCE1 in the meiosis. Thus, the spermatogenesis was completely arrested at pachytene stage in this patient. Altogether, CNVs in *SYCE1* caused meiotic arrest and NOA in these two patients.

## Conclusions

In conclusion, we identified two novel CNVs within *SYCE1* in two idiopathic NOA-affected patients. The meiotic arrest phenotype was ascertained in these two patients via H&E and IF staining. Thus, our study expands the knowledge of variants in *SYCE1* and provides a new insight to understand the genetic etiology of NOA. Further studies are warranted to better understand the mechanism of CNV derivation in *SYCE1* locus.

## Supplementary Information

**Additional file 1**. CNV analysis using WES data and observation of wild- and mutated-type SYCE1 expression in vitro. (**A**–**C**) CNV analysis in the NOA-affected patient (P6326) (**A**) and his parents (**B**–**C**) using WES data; (**D**) WB assay detected the expression and size of mutant SYCE1 protein. The protein molecular weight represents the fusion expression of SYCE1 and Flag. NC indicated the negative control.

## Data Availability

The patients’ detected variants have been submitted to the Dryad (https://datadryad.org/stash). The dataset was assigned a unique identifier (10.5061/dryad.bk3j9kddj).

## Abbreviations

WES: Whole-exome sequencing
NOA: Non-obstructive azoospermia
CNV: Copy number variation
LoF: Loss of function
H&E: Hematoxylin and eosin
IF: Immunofluorescence
SCOS: Sertoli cell only syndrome
MA: Maturation arrest
HS: Hypo-spermatogenesis
RBMY: RNA binding motif protein X-linked
SNVs: Single-nucleotide variants
DMC1: DNA meiotic recombinase 1
STAG3: Stromal antigen 3
TEX11: Testis expressed 11
SHOC1: Shortage in chiasmata 1
SYCE1: Synaptonemal complex central element protein 1
MEIOB: Meiosis specific with OB-fold
CCDC155: Coiled-coil domain containing 155
TEX14: Testis expressed 14
TEX15: Testis expressed 15
XRCC2: X-ray repair cross complementing 2
PCR: Polymerase chain reaction
SYCP3: Synaptonemal complex protein 3
DMC1: DNA meiotic recombinase 1
PBS-T: Phosphate buffer saline-Tween
DAPI: 4′,6-diamidino-2-phenylindole
mTESE: Microsurgical testicular sperm extraction
SYCE3: Synaptonemal complex central element protein 3
SIX6OS1: Six6 opposite strand transcript 1
SYCE2: Synaptonemal complex central element protein 2
TEX12: Testis expressed 12
ACMG/AMP: American College of Medical Genetics and Genomics and The Association for Molecular Pathology
