# Supplementary material for: Novel copy number variations within SYCE1 caused meiotic arrest and non-obstructive azoospermia
Source: BMC Med Genomics. 2022 Jun 19;15:137. doi: 10.1186/s12920-022-01288-8 (PMC9208180; doi:10.1186/s12920-022-01288-8)

**Methods**

**Western blot**

To obtain cell lysates, HEK293T cells were transfected with 3×Flag-SYCE1-WT or 3×Flag-SYCE1-MUT, respectively. 72 hours later, total proteins were extracted from the cultured cells homogenized in RIPA lysis buffer (Santa Cruz). Thirty micrograms of proteins were electrophoresed on the SDS-PAGE and transferred to polyvinylidene difluoride membranes. After blocking with 5% milk, membranes were incubated with antibodies against Flag (Sigma Aldrich, F1804, dilution 1: 1000) and GAPDH (Proteintech, 60004, dilution 1: 2000) at 4°C overnight. Membranes were incubated with secondary antibodies for 1 h at room temperature. After incubation with the corresponding secondary antibodies, the blots were detected by the enhanced chemiluminescence (Chemi-Doc XRS, Bio-Rad).

**Supplementary Fig 1** **CNV analysis using WES data and observation of wild- and mutated-type SYCE1 expression in vitro.**

(A-C) CNV analysis in the NOA-affected patient (P6326) (A) and his parents (B-C) using WES data; (D) WB assay detected the expression and size of mutant SYCE1 protein. The protein molecular weight represents the fusion expression of SYCE1 and Flag. NC indicated the negative control.


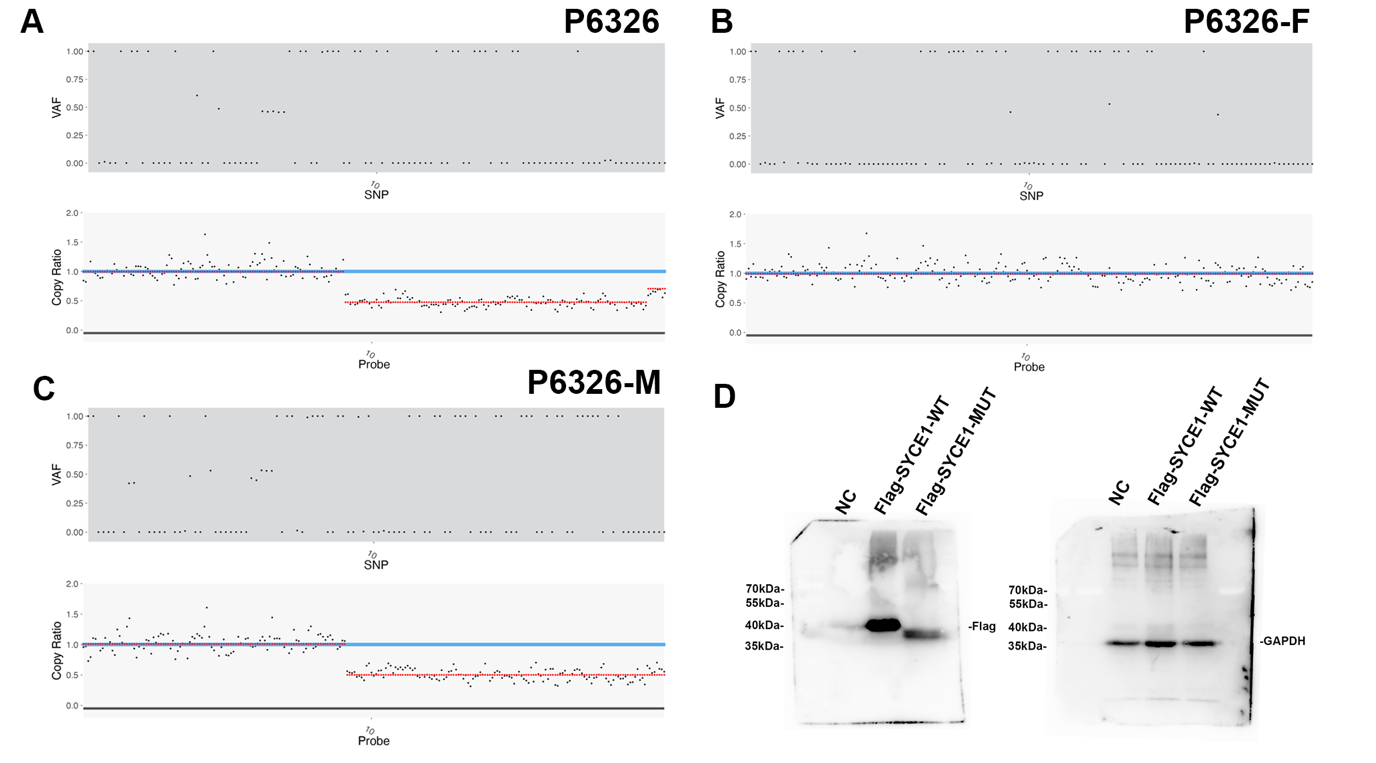

Supplement: Supplementary file 1 — Additional file 1. CNV analysis using WES data and observation of wild- and mutated-type SYCE1 expression in vitro. (A–C) CNV analysis in the NOA-affected patient (P6326) (A) and his parents (B–C) using WES data; (D) WB assay detected the expression and size of mutant SYCE1 protein. The protein molecular weight represents the fusion expression of SYCE1 and Flag. NC indicated the negative control. [file 12920_2022_1288_MOESM1_ESM.docx]
